# Supplementary material for: The Identification of the Mitochondrial DNA Polymerase γ (Mip1) of the Entomopathogenic Fungus Metarhizium brunneum
Source: Microorganisms. 2024 May 23;12(6):1052. doi: 10.3390/microorganisms12061052 (PMC11205540; doi:10.3390/microorganisms12061052)
Supplement: Supplementary file 1 [file microorganisms-12-01052-s001.zip › microorganisms-3009752-supplementary.pdf]

Supplementary data – Varassas et al.

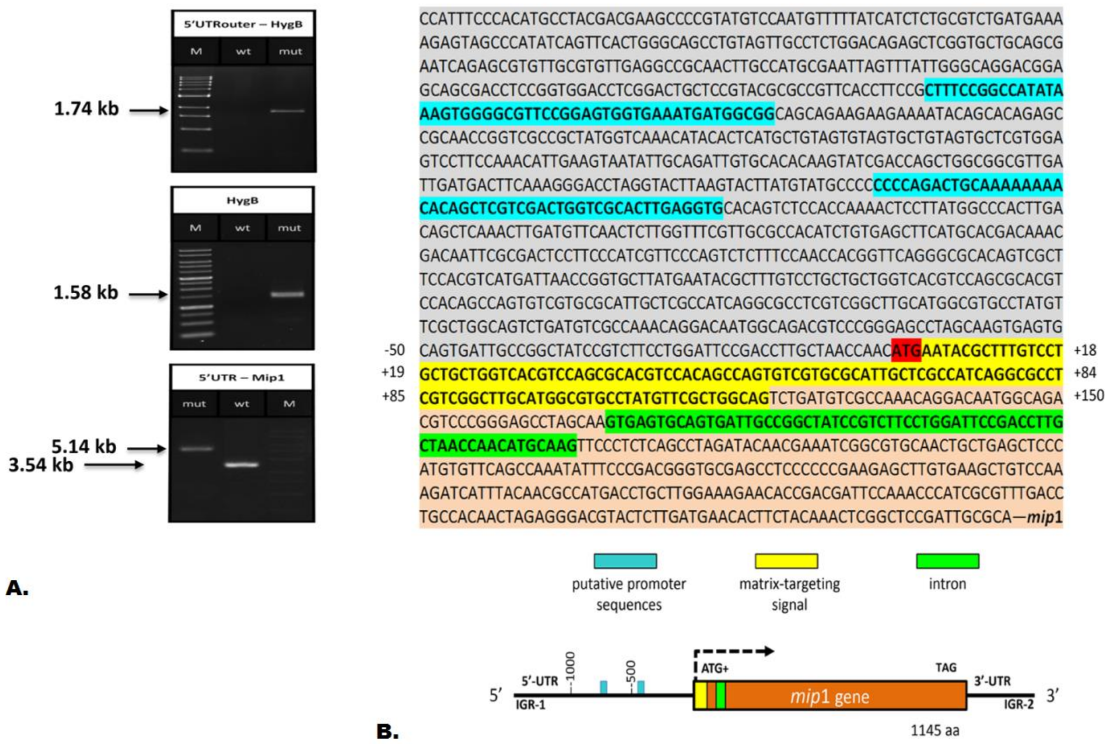

Supplementary Figure S1: The mip1 gene structure of *M. brunneum* and verification strategy of constructing the mutant mip1$^-$ strain A) Replacement-specific PCR analysis. Confirmation of the predicted gene targeting conducted with primer combinations that only amplify a signal in the recombinant locus (mutant). The absence of a WT-specific signal in the clonal mutants Mip1$^-$ (Δ1) and plasmid pHygB-Mip1 (P) confirms the genetic homogeneity of the mutant isolate. B) Promoter region of *mip*1. The 1-kb promoter region of the *M. brunneum* ARSEF 3297 *mip*1 gene contains a cluster of two putative promoters (blue color). aa, amino acids.

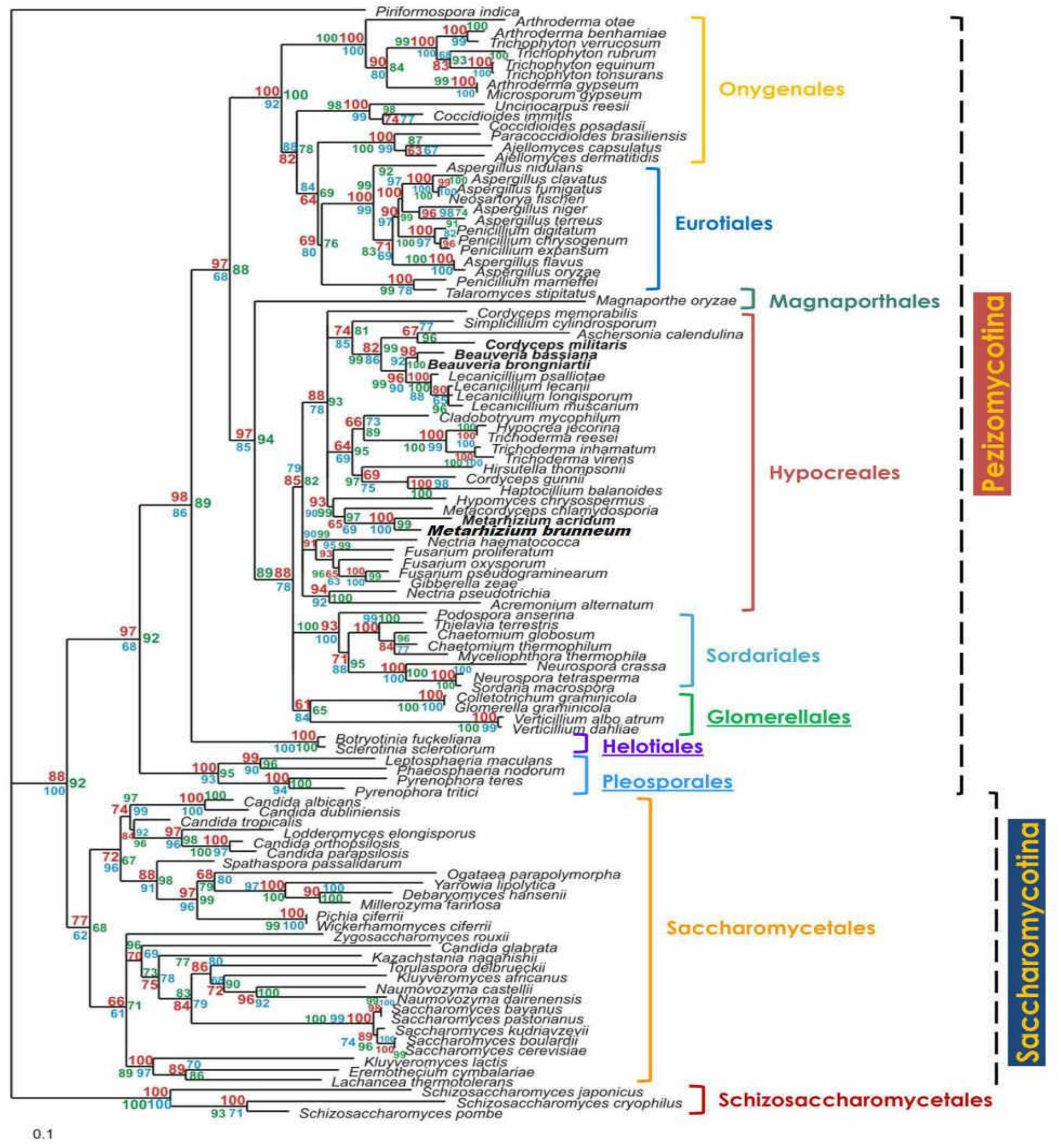

Piriformospora indica
Arthroderma otae
Arthroderma benhamiae
Trichophyton verrucosum
Trichophyton rubrum
Trichophyton equinum
Trichophyton tonsurans
Arthroderma gypseum
Microsporum gypseum
Uncinocarpus reesii
Coccidioides immitis
Coccidioides posadasii
Paracoccidioides brasiliensis
Ajellomyces capsulatus
Ajellomyces dermatitidis
Onygenales
Aspergillus nidulans
Aspergillus clavatus
Aspergillus fumigatus
Neosartorya fischeri
Aspergillus niger
Aspergillus terreus
Penicillium digitatum
Penicillium chrysogenum
Penicillium expansum
Aspergillus flavus
Aspergillus oryzae
Penicillium marneffei
Talaromyces stipitatus
Eurotiales
Magnaporthe oryzae
Magnaporthales
Cordyceps memorabilis
Simplicillium cylindrosporum
Aschersonia calendulina
Cordyceps militaris
Beauveria bassiana
Beauveria brongniartii
Lecanicillium psalliotae
Lecanicillium lecanii
Lecanicillium longisporum
Lecanicillium muscarium
Cladobotryum mycophilum
Hypocrea jecorina
Trichoderma reesei
Trichoderma inhamatum
Trichoderma virens
Hirsutella thompsonii
Cordyceps gunnii
Haptocillium balanoides
Hypomyces chrysospermus
Metacordyceps chlamydosporia
Metarhizium acridum
Metarhizium brunneum
Nectria haematococca
Fusarium proliferatum
Fusarium oxysporum
Fusarium pseudograminearum
Gibberella zeae
Nectria pseudotrichia
Acremonium alternatum
Hypocreales
Podospora anserina
Thielavia terrestris
Chaetomium globosum
Chaetomium thermophilum
Myceliophthora thermophila
Neurospora crassa
Neurospora tetrasperma
Sordaria macrospora
Sordariales
Colletotrichum graminicola
Glomerella graminicola
Verticillium albo atrum
Verticillium dahliae
Glomerellales
Botryotinia fuckeliana
Sclerotinia sclerotiorum
Helotiales
Leptosphaeria maculans
Phaeosphaeria nodorum
Pyrenophora teres
Pyrenophora tritici
Pleosporales
Pezizomycotina
Candida albicans
Candida dubliniensis
Candida tropicalis
Lodderomyces elongisporus
Candida orthopsilosis
Candida parapsilosis
Spathaspora passalidarum
Ogataea parapolymorpha
Yarrowia lipolytica
Debaryomyces hansenii
Millerozyma farinosa
Pichia ciferrii
Wickerhamomyces ciferrii
Zygosaccharomyces rouxii
Candida glabrata
Kazachstania naganishii
Torulaspora delbrueckii
Kluyveromyces africanus
Naumovozyma castellii
Naumovozyma dairenensis
Saccharomyces bayanus
Saccharomyces pastorianus
Saccharomyces kudriavzevii
Saccharomyces boulardii
Saccharomyces cerevisiae
Kluyveromyces lactis
Eremothecium cymbalariae
Lachancea thermotolerans
Saccharomycetales
Saccharomycotina
Schizosaccharomyces japonicus
Schizosaccharomyces cryophilus
Schizosaccharomyces pombe
Schizosaccharomycetales
0.1

A.

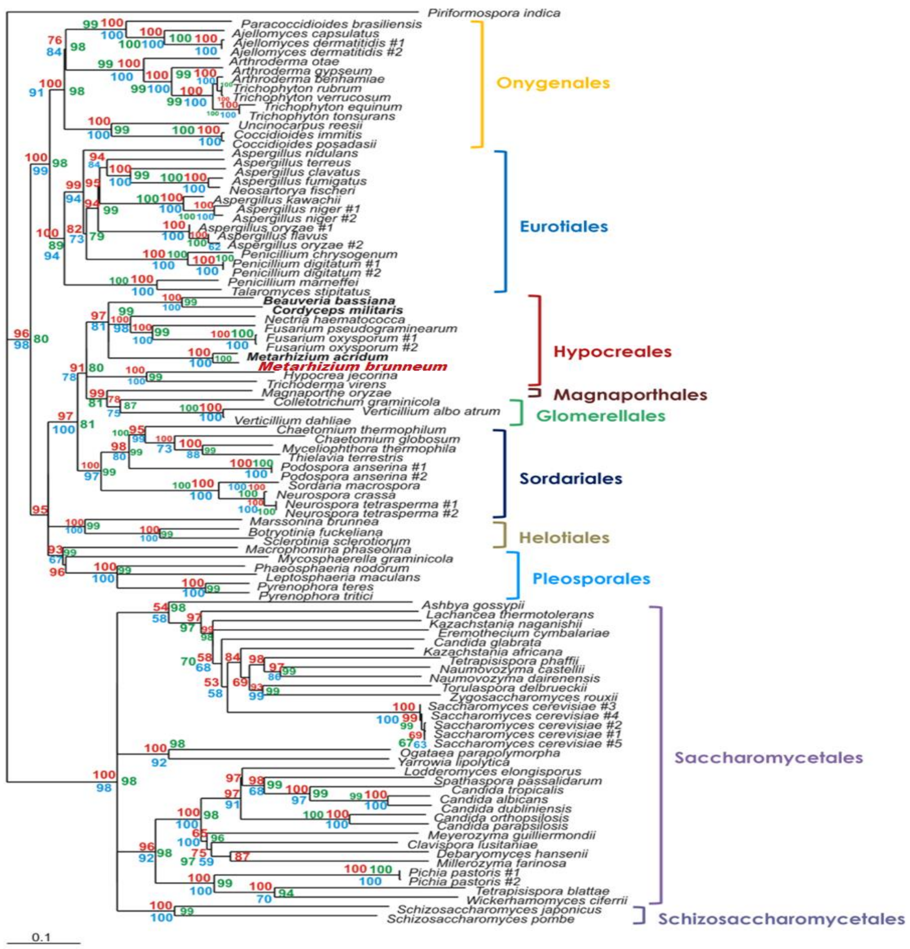

B.

Supplementary Figure S2: **The phylogenetic analysis**. A) The phylogenetic tree of the *mip*1 matrix of representative fungal species from the whole Kingdom is produced by Neighbor Joining analysis and in accordance (100%) to the tree of Bayesian method. Support of clades is provided with bootstrap values (NJ and MP bootstrap in red and blue color, respectively) and Posterior Probabilities (PP) of the Bayesian method (PP in green color). B) The phylogenetic tree of selected ascomycetes constructed based on ITS1-5.8S-ITS2 gene sequences (Supplementary Table S1). Bootstrap support was calculated from 10000 replicates (NJ – red numbers), 1000 replicates (MP – blue numbers) and Posterior Probability (BI – green numbers), provided with PAUP and MrBayes. B) The phylogenetic tree of ITS (internal transcribed spacer) regions of different species of ascomycetes were collected to create matrices for phylogenetic trees (Table S1). The sequences were aligned by Lasergene's MegAlign v.11 program (Burland, 2000) using the ClustalV method with default settings. The phylogenetic tree constructions were performed using Neighbor Joining (NJ), Maximum Parsimony (MP) and Bayesian Inference (BI) methods through PAUP4 (Wilgenbusch et al., 2003) and MrBayes (Ronquist et al., 2003) software, respectively. For NJ analyses, reliability of nodeswas evaluated using 10.000 bootstrap iterations for individual datasets. For BI analyses, the determination of the evolutionary model, which was best suitable for each

dataset, was performed using the program JmodelTest (ver. 2.0) (Guindon and Gascuel, 2003; Darriba et al., 2012) for the ITS and Mip1 matrices respectively. The BIC Information Criterion was applied, and the best nucleotide substitution model was found. In detail, GTR+G and GTR+I were applied for the ITS dataset and for the *mip*1 dataset, respectively. Additionally, two independent MCMCMC analyses were performed, using 10 million generations and sampling set adjustment for every 100.000 generations. The remaining parameters were set to default in all cases.

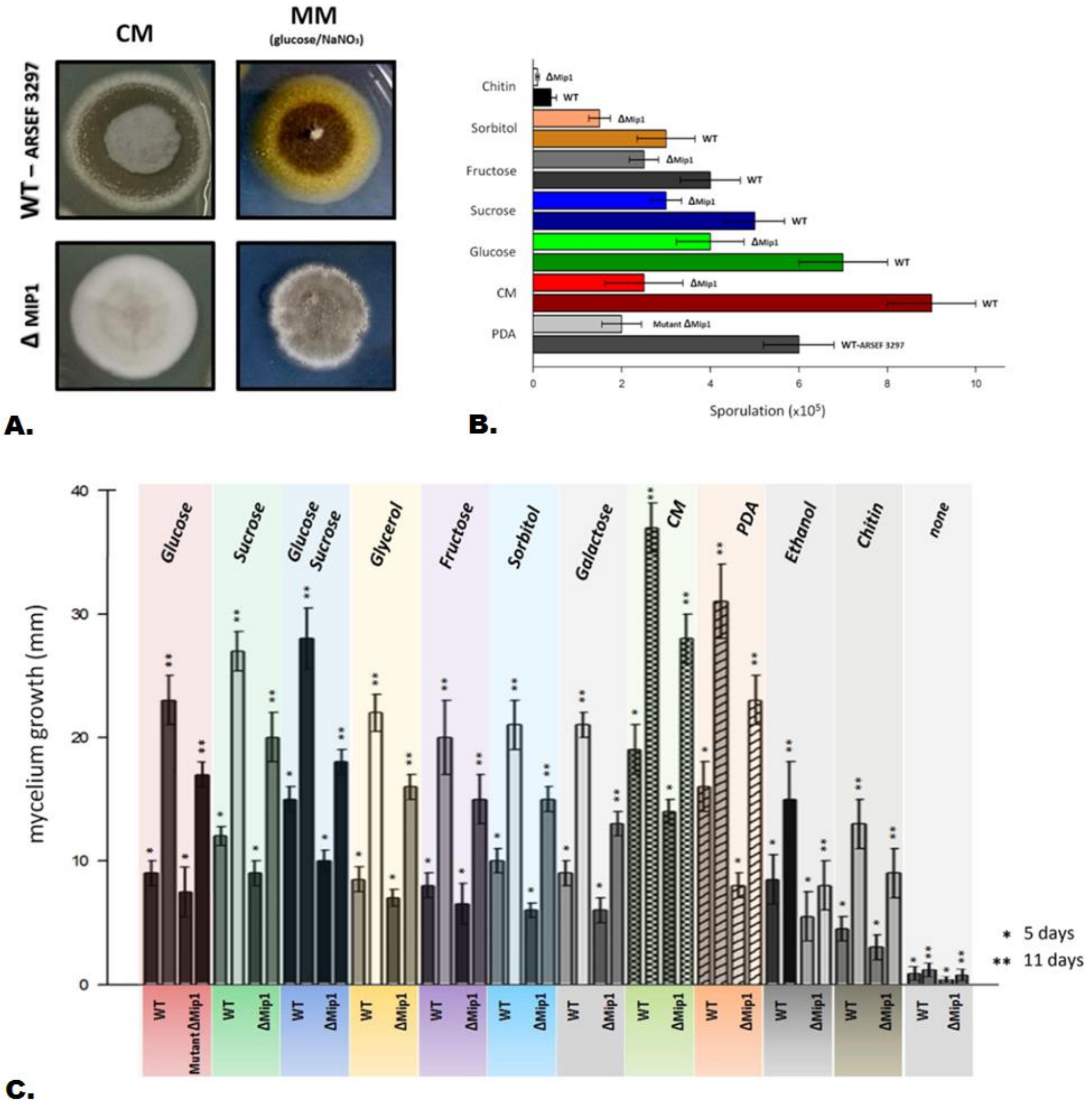

Supplementary Figure S3: **Role of Mip1 in Growth and Physiology**. A) Growth of the WT, ΔMip1 strains on CM and MM media. B) Abundance of conidial production of the two strains. C) Effects of different carbon sources at a concentration of 1 % (w/v) on the growth of WT and ΔMip1 strains after 5 and 11 days at 25 °C on MM. All experiments were performed in triplicate. Bars: SD. Statistical significance of differences was tested by one-way ANOVA, followed by Tukey's post hoc test ($p \leq 0.001$).

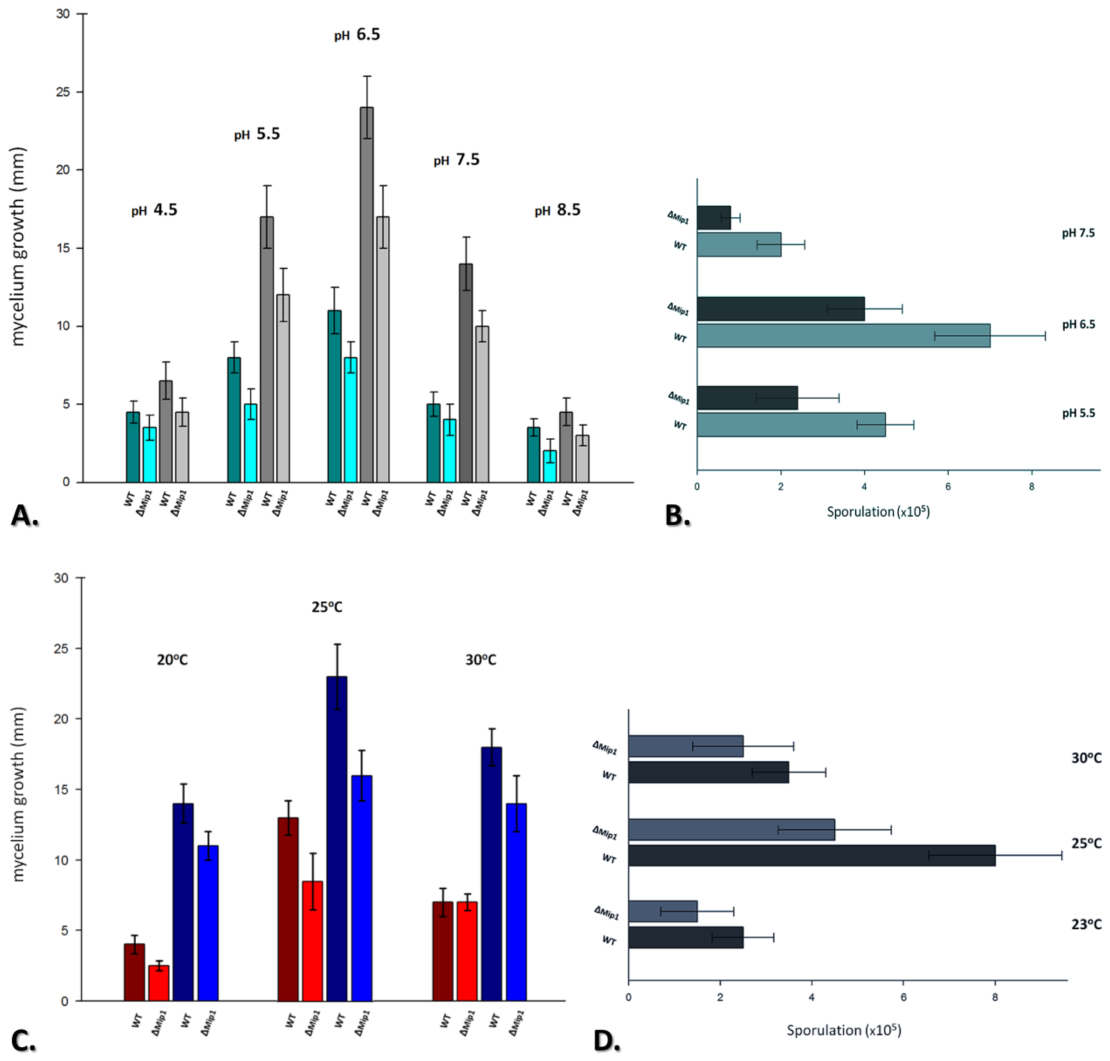

Supplementary Figure S4: **Disruption of Mip1 weakens tolerance to temperature and pH variation and affects hyphal growth and sporulation**. A, B, C, D) Effect of pH and temperature on the hyphal growth and sporulation of two strains. All fungal strains were grown on MM for 5 (pH:cyan/T:red) and 11 (pH:grey/T:bleu) days [each condition was tested in triplicate, bars = SD, statistical testing by one-way ANOVA followed by Tukey's post hoc test ($p \leq 0.001$)].

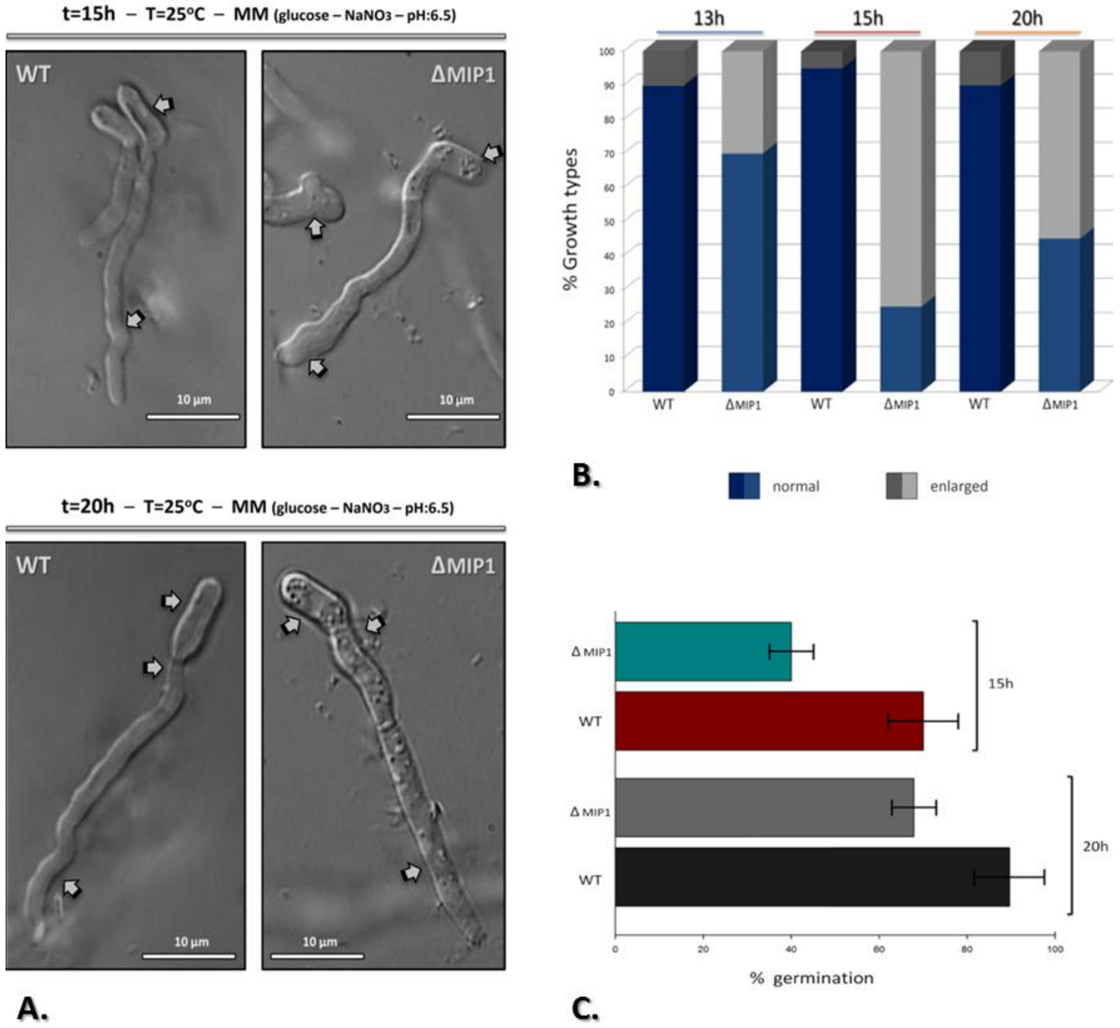

Supplementary Figure S5: **Disruption of *mip*1 Affected Hyphal Growth**. A) Microscopic morphology of hyphal cells at the early stages of germination (mother cell/conidium and germ tube) at 25 ^{o}C, for 15 and 20 h of WT and ΔMip1 strains, respectively. Differences in size and morphology of conidia and hyphal cells of *M. brunneum* grown at different times are marked with arrows. B) Representative types of morphological phenotypes of WT and mutant (normal hyphal cells ─ blue, enlarged hyphal cells ─ grey) for 13, 15, 20 h respectively. Analysis of n=150 germlings of WT and ΔMip1 strains at 25 ^{o}C, respectively. C) Ability of conidia to germinate of the two strains for 15 and 20 hours on MM (25 ^{o}C, pH=6.5). All experiments were performed in triplicate, and 150 conidia were tested for germination per replicate. Bars: SD. Statistical significance of differences was tested by one-way ANOVA, followed by Tukey's post hoc test ($p \leq 0.001$).

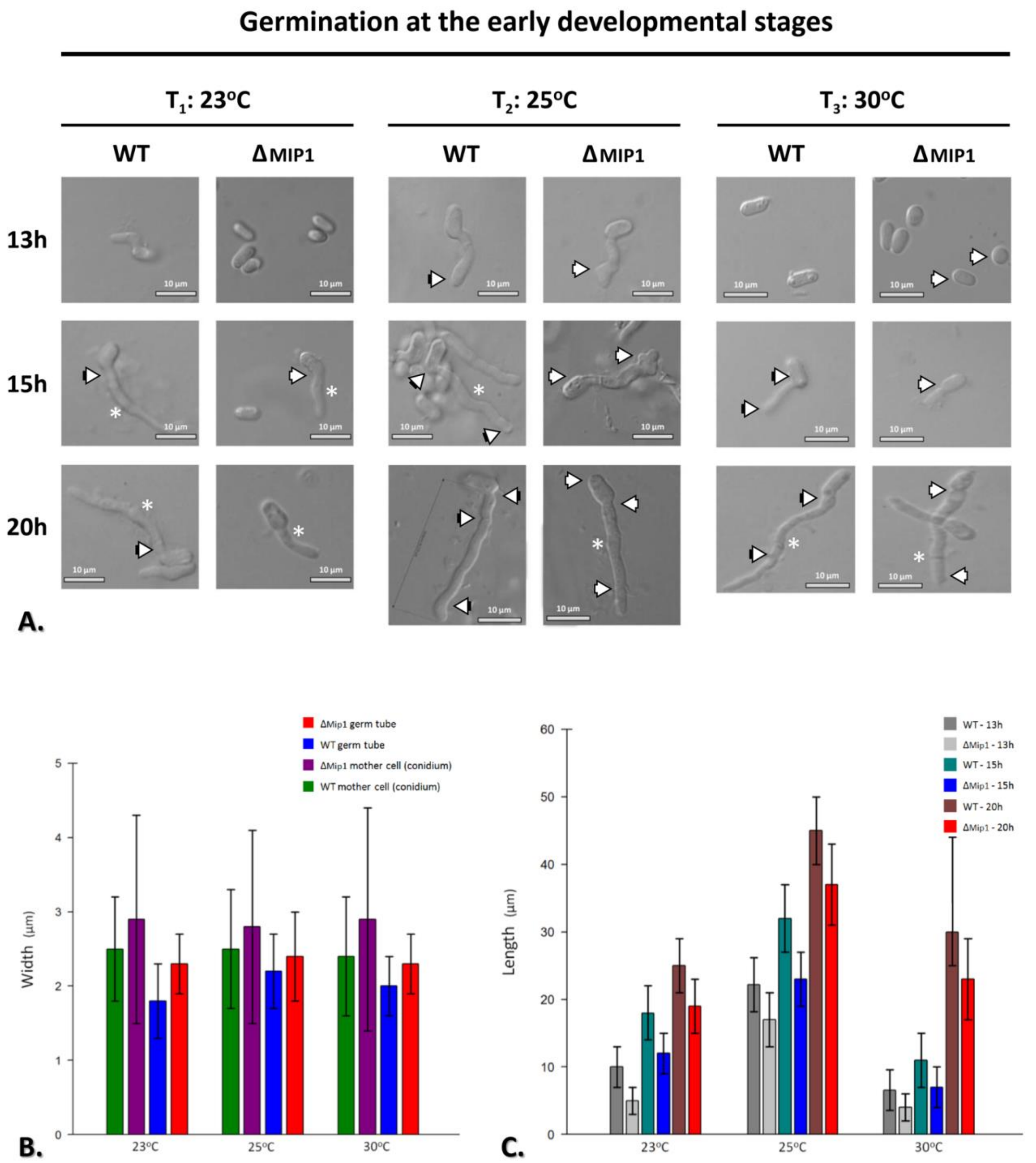

Supplementary Figure S6: **Germination of conidial cells of WT and Mip1⁻ mutant strains at the early developmental stages**. A) Light microscopies showing the dormant (conidia) and germinated conidial cells of *M. brunneum* ARSEF 3297 after 13, 15, and 20 hours of incubation in MM medium at various temperatures (23, 25, 30 ^{o}C) respectively. Differences in size and morphology of conidia and hyphal cells of *M. brunneum* grown under different parameters (time, temperature) are marked with arrowheads and asterisks. B,C) Conidial length and width (µm) of WT and ΔMip1 mutant strains at the early developmental stages. Each strain was tested in triplicate, and 150 conidia were analyzed per replicate. Bars = SD. Statistical significance of differences was tested by one-way ANOVA, followed by Tukey's post-hoc test ($p \leq 0.05$).

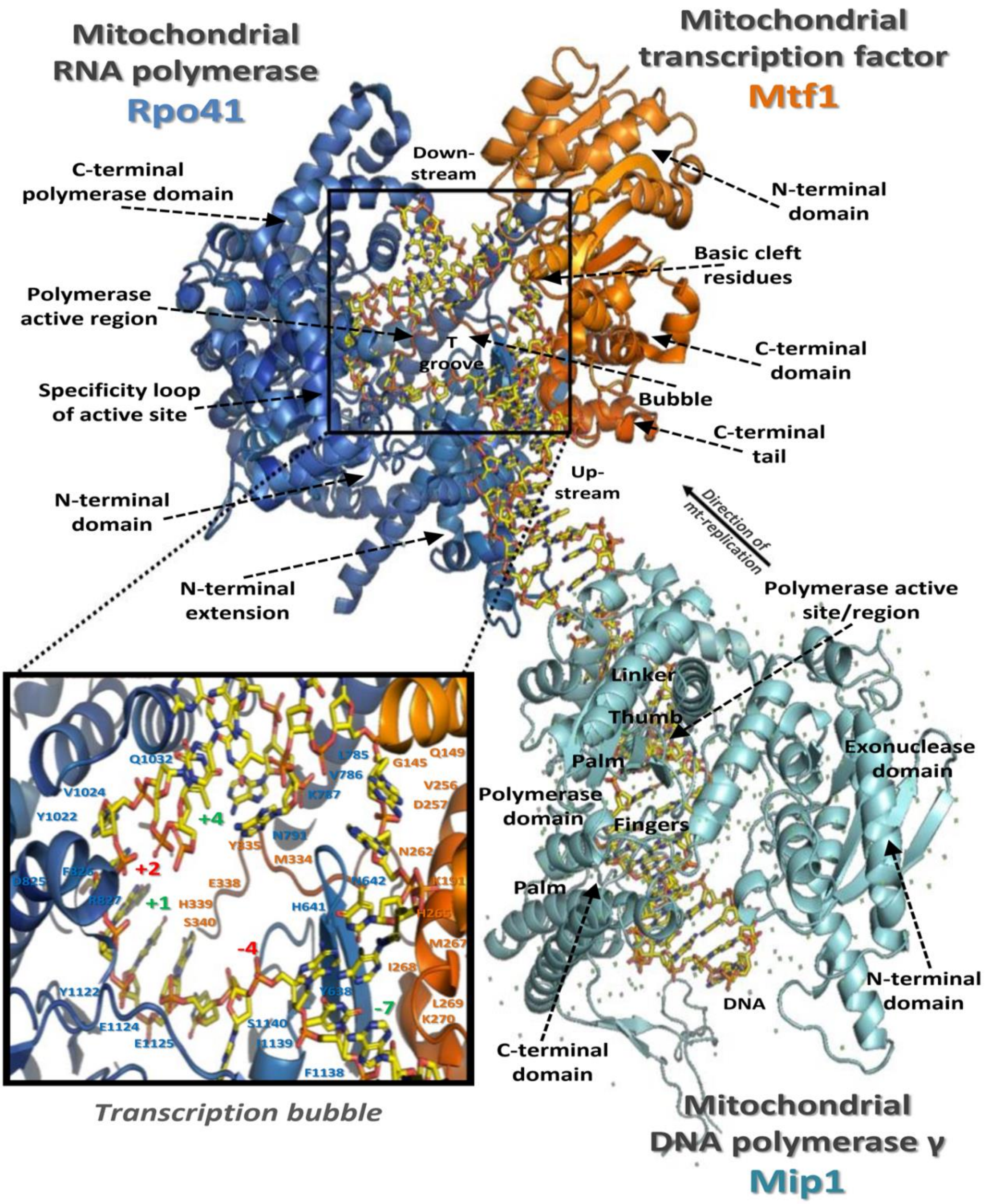

Supplementary Figure S7: **The combined structure of Mip1-Rpo41-Mtf1 holoenzyme for initiation of mtDNA replication**. The sc-mtRNA polymerase (blue) (PDB_ 6YMW) is associated with the RNA polymerase of bacteriophage T7 (PDB_1QLN), while the transcriptional sc-mtTFB agent (orange) shows some similarity to the amino acid residues of the bacterial agent "σ". These two proteins form a complex between them (open complex), which is able to initiate transcription by interacting with the promoter sequence *in vitro* (transcription bubble). A schematic representation of the interactions of the transcription bubble with surrounding protein residues of MTF1 (orange) and y-mtRNAP (blue). Domains of Mip1 (*M. brunneum* ARSEF 3297 - cyan) as determined after bioinformatics analysis in this study are shown.

Mechanism of mitochondrial DNA replication

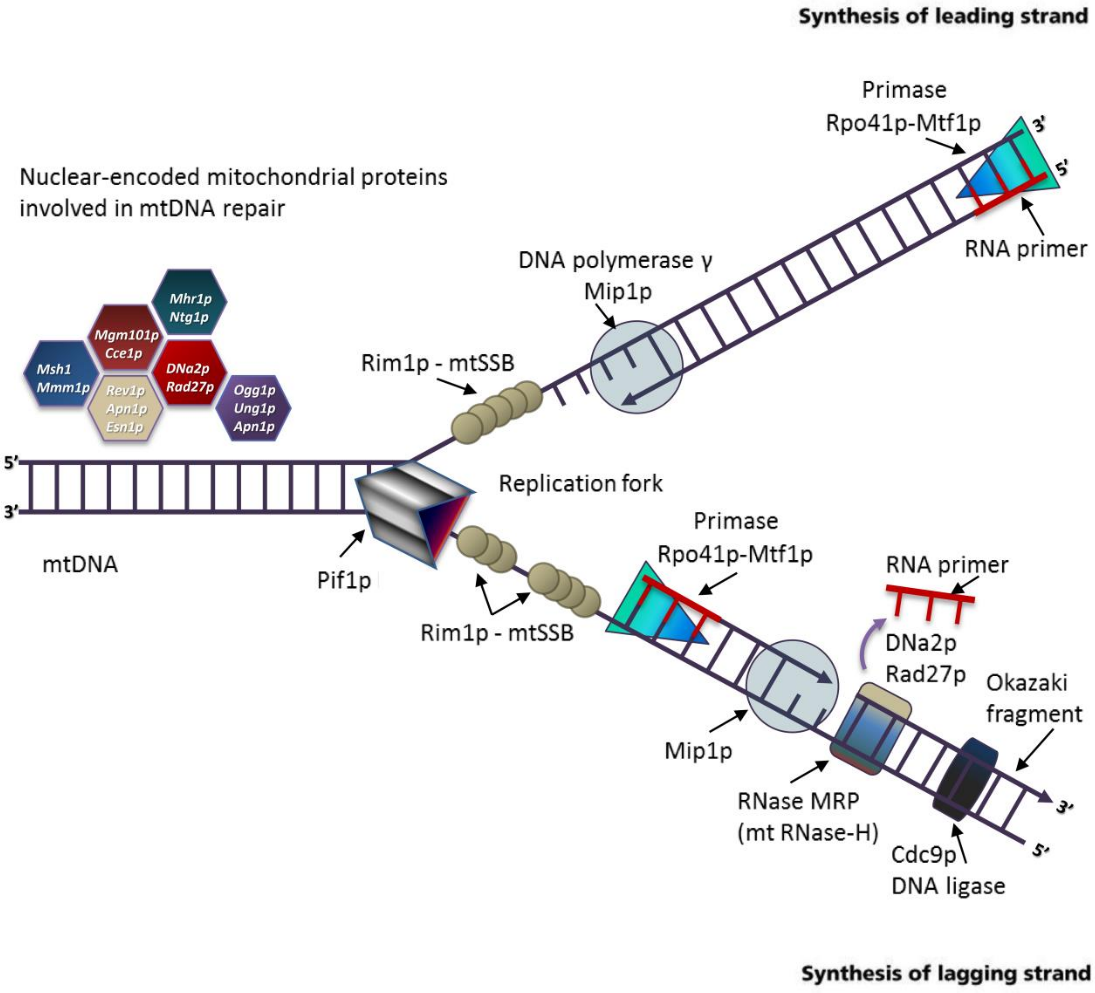

Supplementary Figure S8: ***In silico* model of mitochondrial replication** in Ascomycetes (rolling circle replication) based on literature from the yeast *S. cerevisiae*. Initially, at the start of mitochondrial replication at ori/rep regions, the mtDNA is denatured, the starting positions are uncovered and the DNA helicase Pif1 is conscripted, beginning to unravel the mtDNA to form two local replication forks. Simultaneously, both in continuous and in discontinuous strands, specific proteins Rim1 (mtSSB) are bound to ss-mtDNA to stabilize the latter, followed by the placement of Mip1 DNA polymerase γ on mitochondrial genome. However, RNA primers are removed by the RNase MRP, assisted by the nucleases DNa2 and Rad27. Then, the DNA polymerase γ (Mip1) “fills” this gap, and the adjacent fragments of mtDNAare ligated by the DNA ligase Cdc9. A Topoisomerase (gyrase) has not been found in *M. brunneum* mitochondria yet.

Supplementary Table S1: The fungi used in the phylogenetic analysis of this study, their taxonomy and the GenBank accession numbers of their ITS1-5.8S-ITS2 rDNA and *mip*1 gene regions used in the respective gene matrices.

Species name	Strain	Subphylum	Class	Order	ITS	*mip*1
Macrophomina phaseolina	Mp001NY12	Pezizomycotina	Dothideomycetes	Botryosphaeriales	KC800709 581 bp	XM_001800707 3393 bp
Mycosphaerellagraminicola	T48	Pezizomycotina	Dothideomycetes	Capnodiales	AF181694 572 bp	XM_003854842 3504 bp
Aspergillus clavatus	DAOM 2163	Pezizomycotina	Eurotiomycetes	Eurotiales	JN942917 556 bp	XM_001274551 3405 bp
Aspergillus flavus	M 1184/05	Pezizomycotina	Eurotiomycetes	Eurotiales	DQ683124 575 bp	XM_002381150 3048 bp
Aspergillus fumigatus	BAU-1	Pezizomycotina	Eurotiomycetes	Eurotiales	KC142152 597 bp	XM_748296 3408 bp
Aspergillus nidulans	wb260	Pezizomycotina	Eurotiomycetes	Eurotiales	AF455505 579 bp	XM_652552 3405 bp
Aspergillus niger	WM 06.107	Pezizomycotina	Eurotiomycetes	Eurotiales	EF567981 557 bp	XM_001401242 3408 bp
Aspergillus oryzae	P6B2	Pezizomycotina	Eurotiomycetes	Eurotiales	JX429926 567 bp	XM_001824054 3324 bp
Aspergillus terreus	WM 03.218	Pezizomycotina	Eurotiomycetes	Eurotiales	EF568102 566 bp	XM_001211183 3396 bp
Neosartorya fischeri	KACC 41182	Pezizomycotina	Eurotiomycetes	Eurotiales	JN943576 505 bp	XM_001259433 3330 bp
Penicillium chrysogenum	DAOM 2137	Pezizomycotina	Eurotiomycetes	Eurotiales	JN942859 541 bp	XM_002567421 3429 bp
Penicillium digitatum	DAOM 2260	Pezizomycotina	Eurotiomycetes	Eurotiales	JN942856 542 bp	XM_001274551 3405 bp
Penicillium expansum	DAOM 2150	Pezizomycotina	Eurotiomycetes	Eurotiales	JN942855 540 bp	
Penicillium marneffei	SUMS0351	Pezizomycotina	Eurotiomycetes	Eurotiales	FJ009562 556 bp	XM_002146756 3671 bp
Sclerotinia sclerotiorum	PSHB1	Pezizomycotina	Eurotiomycetes	Eurotiales	KF791510 564 bp	XM_001587550 3441 bp
Talaromyces stipitatus	MJD10-4	Pezizomycotina	Eurotiomycetes	Eurotiales	JX077005 506 bp	XM_002479033 3390 bp
Colletotrichum graminicola	TC2-028	Pezizomycotina	Sordariomycetes	Glomerellales	AF059676 581 bp	XM_006672692 3399 bp
Glomerella graminicola	99003	Pezizomycotina	Sordariomycetes	Glomerellales	AF289234 526 bp	
Verticillium albo-atrum	Ir3	Pezizomycotina	Sordariomycetes	Glomerellales	AY536046 452 bp	XM_003003228 2892 bp
Verticillium dahliae	MD73	Pezizomycotina	Sordariomycetes	Glomerellales	AF364000 452 bp	DS572705 3135 bp
Botryotinia fuckeliana	LGM002	Pezizomycotina	Leotiomycetes	Helotiales	KC683713 541 bp	XM_001550115 3369 bp
Marssonina brunnea	MB_m1	Pezizomycotina	Leotiomycetes	Helotiales	JN172909 610 bp	XM_001550115 3369 bp
Acremonium alternatum	GL0801	Pezizomycotina	Sordariomycetes	Hypocreales	JX241639 540 bp	
Aschersonia calendulina	SM00186.01	Pezizomycotina	Sordariomycetes	Hypocreales	JN942615 609 bp	
Beauveria bassiana	Bb3167	Pezizomycotina	Sordariomycetes	Hypocreales	AF322932 495 bp	XM_006672692 3399 bp
Beauveria brongniartii	IBCB548	Pezizomycotina	Sordariomycetes	Hypocreales	KC004067 498 bp	
Cladobotryum mycophilum	CL80	Pezizomycotina	Sordariomycetes	Hypocreales	EU340834 488 bp	
Cordyceps confragosa	UB-10823	Pezizomycotina	Sordariomycetes	Hypocreales	KF225043 566 bp	
Cordyceps gunnii	ARSEF 6828	Pezizomycotina	Sordariomycetes	Hypocreales	HM14063 503 bp	
Cordyceps memorabilis	CCRC 32218	Pezizomycotina	Sordariomycetes	Hypocreales	GQ461583 551 bp	

Cordyceps militaris	**NBRC 9787**	**Pezizomycotina**	**Sordariomycetes**	Hypocreales	JN943433 555 bp	XM_006672692 3399 bp
Fusarium oxysporum	**ML-5-2**	**Pezizomycotina**	**Sordariomycetes**	Hypocreales	AY387705 445 bp	XM_385754 3386 bp
Fusarium proliferatum	**CATASMmd9**	**Pezizomycotina**	**Sordariomycetes**	Hypocreales	GU074010 519 bp	
Fusarium pseudogr/rum	**WZ-8A**	**Pezizomycotina**	**Sordariomycetes**	Hypocreales	JN862235 517 bp	XM_385692 3375 bp
Gibberella zeae	**YS-114**	**Pezizomycotina**	**Sordariomycetes**	Hypocreales	HQ671191 505 bp	
Gliocladium cibotii	**CBS 823.73**	**Pezizomycotina**	**Sordariomycetes**	Hypocreales	AF048739 523 bp	
Haptocillium balanoides	**ARSEF3350**	**Pezizomycotina**	**Sordariomycetes**	Hypocreales	EU086434 532 bp	
Hirsutella thompsonii	**1456**	**Pezizomycotina**	**Sordariomycetes**	Hypocreales	FJ973072 512 bp	
Hypocrea jecorina	**TUB F-1034**	**Pezizomycotina**	**Sordariomycetes**	Hypocreales	AF486007 648 bp	XM_003655195 3825 bp
Hypomyces chrysospermus	**JZB2115018**	**Pezizomycotina**	**Sordariomycetes**	Hypocreales	JQ283968 599 bp	
Lecanicillium lecanii	**ICAL-7**	**Pezizomycotina**	**Sordariomycetes**	Hypocreales	FJ515771 562 bp	
Lecanicillium muscarium	**ARSEF 2323**	**Pezizomycotina**	**Sordariomycetes**	Hypocreales	EF513017 496 bp	
Lecanicillium psalliotae	**CBS 532.81**	**Pezizomycotina**	**Sordariomycetes**	Hypocreales	JN049846 519 bp	
Metacordycepschlamydosporia	**Pcp31**	**Pezizomycotina**	**Sordariomycetes**	Hypocreales	KC291612 582 bp	
Metarhizium acridum	**ARSEF 7486**	**Pezizomycotina**	**Sordariomycetes**	Hypocreales	HQ331458 563 bp	EFY91379 3273 bp
Metarhizium brunneum	**ARSEF-3297**	**Pezizomycotina**	**Sordariomycetes**	Hypocreales	AY387578 538 bp	EFZ00872 3273 bp
Nectria haematococca	**NHGR48**	**Pezizomycotina**	**Sordariomycetes**	Hypocreales	AF455451 582 bp	XM_003048718 3718 bp
Nectria pseudotrichia	**W7061a**	**Pezizomycotina**	**Sordariomycetes**	Hypocreales	GU232860 492 bp	
Pochonia suchlasporia	**NS-17**	**Pezizomycotina**	**Sordariomycetes**	Hypocreales	JQ839272 572 bp	
Simplicilliumcylindrosporum	**JCM 18172**	**Pezizomycotina**	**Sordariomycetes**	Hypocreales	AB604006 606 bp	
Trichoderma inhamatum	**GL0834**	**Pezizomycotina**	**Sordariomycetes**	Hypocreales	JX241670 586 bp	
Trichoderma reesei	**RSPG_24**	**Pezizomycotina**	**Sordariomycetes**	Hypocreales	KC478546 559 bp	
Trichoderma virens	**GL-21**	**Pezizomycotina**	**Sordariomycetes**	Hypocreales	AF099008 636 bp	XM_003048718 3718 bp
Magnaporthe oryzae	**DH37quan3**	**Pezizomycotina**	**Sordariomycetes**	Magnaporthales	GU073121 517 bp	XM_003713894 3981 bp
Ajellomyces capsulatus	**G186AR**	**Pezizomycotina**	**Eurotiomycetes**	Onygenales	AF156892 541 bp	XM_001542338 3381 bp
Ajellomyces dermatitidis	**ATCC 60636**	**Pezizomycotina**	**Eurotiomycetes**	Onygenales	HQ02673 543 bp	XM_002621172 3899 bp
Arthroderma benhamiae	**UAMH 7339**	**Pezizomycotina**	**Eurotiomycetes**	Onygenales	AF170467 837 bp	XM_003010642 3417 bp
Arthroderma gypseum	**M1003 4393**	**Pezizomycotina**	**Eurotiomycetes**	Onygenales	JX101937 615 bp	XM_003170339 3417 bp
Arthroderma otae	**WM 05.15**	**Pezizomycotina**	**Eurotiomycetes**	Onygenales	EF568060 695 bp	XM_002845176 3402 bp
Coccidioides immitis	**CBS 113857**	**Pezizomycotina**	**Eurotiomycetes**	Onygenales	EF186790 539 bp	XM_003067196 3890 bp
Coccidioides posadasii	**S1203408.2**	**Pezizomycotina**	**Eurotiomycetes**	Onygenales	KF373787 872 bp	XM_003067196 3890 bp
Microsporum gypseum	**WM 06.353**	**Pezizomycotina**	**Eurotiomycetes**	Onygenales	EF568061 624 bp	
Paracoccidioidesbrasiliensis	**Pb10**	**Pezizomycotina**	**Eurotiomycetes**	Onygenales	AY374339 566 bp	XM_002795776 3429 bp
Trichophyton	**LM 03**	**Pezizomycotina**	**Eurotiomycetes**	Onygenales	AF170458	XM_003170339

equinum					845 bp	3417 bp
richophyton rubrum	**ATCC 2888**	**Pezizomycotina**	**Eurotiomycetes**	Onygenales	AF170472 850 bp	XM_003230925 3414 bp
Trichophyton tonsurans	**UAMH 8552**	**Pezizomycotina**	**Eurotiomycetes**	Onygenales	AF170479 845 bp	XM_002845176 3402 bp
Trichophyton verrucosum	**bM 132**	**Pezizomycotina**	**Eurotiomycetes**	Onygenales	JX122306 622 bp	XM_003017862 3414 bp
Uncinocarpus reesii	**WM 4487**	**Pezizomycotina**	**Eurotiomycetes**	Onygenales	AJ271566 595 bp	XM_002584259 3423 bp
Arthrobotrys oligospora	**ZO_8576**	**Pezizomycotina**	**Orbiliomycetes**	Orbiliales	JX244893 601 bp	
Leptosphaeria maculans	**CBS 260.94**	**Pezizomycotina**	**Dothideomycetes**	Pleosporales	JF740235 503 bp	XM_003842346 3507 bp
Phaeosphaeria nodorum	**STAN24**	**Pezizomycotina**	**Dothideomycetes**	Pleosporales	AY817686 613 bp	XM_001800707 3393 bp
Pyrenophora teres	**DAOM 2326**	**Pezizomycotina**	**Dothideomycetes**	Pleosporales	JN943645 499 bp	XM_003295798 3474 bp
Pyrenophora tritici	**DAOM 5584**	**Pezizomycotina**	**Dothideomycetes**	Pleosporales	GQ168740 472 bp	XM_001940388 4581 bp
Piriformospora indica	**AFTOL-ID612**	**Agaricomycotina**	**Agaricomycetes**	Sebacinales	DQ411527 547 bp	CAFZ01000502 4343 bp
Chaetomium globosum	**WM 05.9**	**Pezizomycotina**	**Sordariomycetes**	Sordariales	EF568043 533 bp	XM_001229769 3249 bp
Chaetomium thermophilum	**ATCC 16451**	**Pezizomycotina**	**Sordariomycetes**	Sordariales	JF412013 480 bp	XM_006692745 3585 bp
Myceliophthorathermophila	**UAMH 2474**	**Pezizomycotina**	**Sordariomycetes**	Sordariales	HQ724321 490 bp	XM_003663227 3956 bp
Neurospora crassa	**HT-ITV31**	**Pezizomycotina**	**Sordariomycetes**	Sordariales	KF040479 567 bp	XM_952654 4371 bp
Neurospora tetrasperma	**ATCC 4615**	**Pezizomycotina**	**Sordariomycetes**	Sordariales	GU327631 570 bp	AF111068 4887 bp
Podospora anserina	**ATCC 4625**	**Pezizomycotina**	**Sordariomycetes**	Sordariales	GU327641 536 bp	XM_001908081 3468 bp
Sordaria macrospora	**ATCC 4828**	**Pezizomycotina**	**Sordariomycetes**	Sordariales	AF246293 576 bp	XM_003347807 4530 bp
Thielavia terrestris	**SFCF912-13**	**Pezizomycotina**	**Sordariomycetes**	Sordariales	KF313105 574 bp	XM_003655195 3825 bp
Candida albicans	**WM 231**	**Saccharomycotina**	**Saccharomycetes**	Saccharomycetales	EF568101 522 bp	XM_711645 3687 bp
Candida dubliniensis	**WM 03.79**	**Saccharomycotina**	**Saccharomycetes**	Saccharomycetales	EF568000 499 bp	XM_002420892 3990 bp
Candida glabrata	**WM 02.57**	**Saccharomycotina**	**Saccharomycetes**	Saccharomycetales	EF568002 839 bp	XM_445920 3855 bp
Candida orthopsilosis	**LEMIH40**	**Saccharomycotina**	**Saccharomycetes**	Saccharomycetales	KC846140 512 bp	XM_003870650 4092 bp
Candida parapsilosis	**WM 02.95**	**Saccharomycotina**	**Saccharomycetes**	Saccharomycetales	EF568035 478 bp	XM_003870650 4092 bp
Candida qinlingensis	**AS 2.2509**	**Saccharomycotina**	**Saccharomycetes**	Saccharomycetales	AY450917 531 bp	
Candida tropicalis	**WM 233**	**Saccharomycotina**	**Saccharomycetes**	Saccharomycetales	EF568042 493 bp	XM_002548504 3807 bp
Clavispora lusitaniae	**CBS 6936**	**Saccharomycotina**	**Saccharomycetes**	Saccharomycetales	AY321464 344 bp	XM_002614216 2793 bp
Debaryomyces hansenii	**DAOM 2364**	**Saccharomycotina**	**Saccharomycetes**	Saccharomycetales	JN942909 595 bp	XM_460911 4029 bp
Eremothecium cymbalariae	**Ere1574**	**Saccharomycotina**	**Saccharomycetes**	Saccharomycetales	AY046219 576 bp	XM_003646622 3570 bp
Kazachstania hellenica	**D9W2**	**Saccharomycotina**	**Saccharomycetes**	Saccharomycetales	EU075202 696 bp	XM_003957475 3564 bp
Kluyveromyces africanus	**CBS 657.94**	**Saccharomycotina**	**Saccharomycetes**	Saccharomycetales	AY046155 677 bp	
Kluyveromyces lactis	**UWO80-12**	**Saccharomycotina**	**Saccharomycetes**	Saccharomycetales	AY626023 631 bp	
Lachancea thermotolerans	**BBMC7FA20**	**Saccharomycotina**	**Saccharomycetes**	Saccharomycetales	KF735112 637 bp	XM_002552764 3672 bp
Lodderomyces elongisporus	**ATCC 11503**	**Saccharomycotina**	**Saccharomycetes**	Saccharomycetales	HQ876042 550 bp	XM_001523882 2928 bp

Meyerozyma guilliermondii	**WM 02.72**	**Saccharomycotina**	**Saccharomycetes**	Saccharomycetales	EF568008 565 bp	XM_001487478 3630 bp
Millerozyma farinosa	**WM 803**	**Saccharomycotina**	**Saccharomycetes**	Saccharomycetales	EF568067 627 bp	XM_004199864 3810 bp
Naumovozyma castellii	**CBS 4309**	**Saccharomycotina**	**Saccharomycetes**	Saccharomycetales	AY046180 683 bp	XM_003676608 3507 bp
Naumovozyma dairenensis	**CBS 421**	**Saccharomycotina**	**Saccharomycetes**	Saccharomycetales	AY046181 695 bp	XM_003670393 3519 bp
Ogataea parapolymorpha	**NRRL 1982**	**Saccharomycotina**	**Saccharomycetes**	Saccharomycetales	JF756591 649 bp	XM_002492888 3039 bp
Pichia ciferrii	**NRRL 1031**	**Saccharomycotina**	**Saccharomycetes**	Saccharomycetales	FJ153218 564 bp	XM_002492888 3039 bp
Saccharomyces bayanus	**NBRC 0539**	**Saccharomycotina**	**Saccharomycetes**	Saccharomycetales	AY046152 750 bp	
Saccharomyces boulardii	**EM10049**	**Saccharomycotina**	**Saccharomycetes**	Saccharomycetales	FJ433878 852 bp	
Saccharomyces cerevisiae	**W13**	**Saccharomycotina**	**Saccharomycetes**	Saccharomycetales	KC542799 747 bp	NM_001183750 3765 bp
Saccharomyceskudriavzevii	**ZP 629**	**Saccharomycotina**	**Saccharomycetes**	Saccharomycetales	AY046150 753 bp	
Saccharomyces pastorianus	**NRRL Y-1525**	**Saccharomycotina**	**Saccharomycetes**	Saccharomycetales	AY046151 750 bp	
Spathaspora passalidarum	**ATCC 4345**	**Saccharomycotina**	**Saccharomycetes**	Saccharomycetales	FJ623614 529 bp	XM_002420892 3990 bp
Torulaspora delbrueckii	**WM 821**	**Saccharomycotina**	**Saccharomycetes**	Saccharomycetales	EF568083 756 bp	XM_003683409 3936 bp
Wicker/myces ciferrii	**CBS 111**	**Saccharomycotina**	**Saccharomycetes**	Saccharomycetales	HM156508 495 bp	CAIF01000187 3591 bp
Yarrowia lipolytica	**CBS 11593**	**Saccharomycotina**	**Saccharomycetes**	Saccharomycetales	JX561141 315 bp	XM_505619 3141 bp
Zygosacch/myces rouxii	**CEE 34**	**Saccharomycotina**	**Saccharomycetes**	Saccharomycetales	AY225979 619 bp	XM_002496572 3720 bp
Schizosacch/mycescryophilus	**ATCC 4695**	**Taphrinomycotina**	**Schizosac/mycetes**	Schizosac/mycetales	HQ999972 740 bp	
Schizosacch/mycesjaponicus	**NRRL 7172**	**Taphrinomycotina**	**Schizosac/mycetes**	Schizosac/mycetales	AB243296 605 bp	XM_002173821 3180 bp
Schizosacch/myces pombe	**CHFY0201**	**Taphrinomycotina**	**Schizosac/mycetes**	Schizosac/mycetales	EU916982 502 bp	NM_001023016 3917 bp

Supplementary Table S2: Top Evolutionary Couplings (ECs) between Mitochondrial RNA polymerase (Rpo41) of *S. cerevisiae* and Mitochondrial DNA polymerase gamma (Mip1) of *M. brunneum*, as found by the EV couplings server (https://evcouplings.org/, EVcomplex score ≥0.8). It is important to note that predicted amino acids between Rpo41 and Mip1 are located at similar positions on these two organisms.

**Predicted ECs**		
Residue subunit 1	Residue subunit 2	EVcomplex score
**Rpo41**	**Mip1**	
D482	H523	0,948
L480	E522*	0,904
S477	D519*	0,852
R475	T514	0,817

* these amino acids are conserved in the linker region of Mip1, as found by ET-viewer.
